# Spectroscopic, Thermal, Electrochemical, and Antimicrobial Studies of Mononuclear Manganese(II) Ditolyldithiophosphates

**DOI:** 10.1155/2013/261731

**Published:** 2013-07-31

**Authors:** Ruchi Khajuria, Atiya Syed, Sandeep Kumar, Sushil K. Pandey

**Affiliations:** Department of Chemistry, University of Jammu, Baba Saheb Ambedkar Road, Jammu and Kashmir 180 006, India

## Abstract

New complexes of manganese(II) corresponding to [{(ArO)_2_PS_2_}_2_Mn] and [{(ArO)_2_PS_2_}_2_Mn.nL] (Ar = *o*-, *m*-, *p*-CH_3_C_6_H_4_ and *p*-Cl-*m*-CH_3_C_6_H_3_; *n* = 1, L = N_2_C_12_H_8_, N_2_C_10_H_8_; *n* = 2, L = NC_5_H_5_, P(C_6_H_5_)_3_) have been synthesized and characterized by microelemental analyses (C, H, and N), magnetic susceptibility, molar conductance, thermogravimetric, cyclic voltammetry, and spectral analyses including ESI mass spectrometry, IR, and UV-visible. The presence of a four-and-six coordinated Mn atoms has been established in the complexes and adducts, respectively. Antimicrobial screening of the complexes against gram negative bacteria *E. coli*, *K. pneumonia,* and *P. aeruginosa* and fungus *S. rolfsii* has shown potential bioactivity.

## 1. Introduction

Manganese has attracted the attention of several researchers owing to the fascinating physical and biochemical characteristics. The utility of manganese is quite diversified such as an alloying agent for aluminium, in dry cell batteries (carbon-zinc Leclanche type), oxidizing agent, and so forth [1]. In nature, manganese is present at the active site of a wide range of enzymes such as catalase and ribonucleotide reductase and participates in a variety of biological reactions [2]. Manganese(II) in its coordination compounds exhibits marked preference for hard donor atoms such as oxygen and nitrogen. Manganese(II) complexes containing soft donor atoms are scanty in number as compared with those of other transition metals, presumably due to difficulties encountered in preparing and preserving such complexes [3]. The first evidence for manganese binding to sulfur donor group in metalloprotein is tryptophan-modified Mn(III) containing acid phosphatise [4]. As compared to the other transition metals, manganese forms complexes with 1,1-dithio and 1,2-dithiolene ligands such as [Mn(SPh)_4_]^2−^, [MnCl(SPh)_3_]^2−^, [Mn_4_(SPh)_10_]^2−^, [Mn(edt)_2_]^2−^, [Mn_2_(edt)_4_]^2−^ (edt = ethane-1,2-dithiolate), and [Mn_2_(S_2_-O-xyl)_2_X_2_]^2−^ (X = PhS^−^, Et_2_NCS_2_^−^; X_2_ = S_2_-O-xyl^2−^) [5].

The high efficacy of the ligands containing sulfur atoms has been well established in metal chelation therapy and has high potency as fungicides and pesticides [6]. Among sulfur donor ligands, *O,O*′-dialkyl and alkylene dithiophosphates have been used for producing potential coordination compounds with most of the metals [7–10]. These soft donor ligands are versatile ligands which show both monodentate [11, 12]and bidentate [13–18] behaviour and form complexes with various metal ions, mainly transition metals [16–18]. Various dithiophosphate derivatives find extensive applications in agriculture [19], industries [20, 21], analytical studies [22], and tribology [23–25]. The versatility of applications of dithiophosphates and their ability to combine with metal ions into a variety of coordination complexes have led to a large interest in their structure and the factors governing the properties of dithiophosphate complexes. The best known complexes of manganese with sulfur ligands are dithiophosphinates [26, 27] and the dithiocarbamates [28]. A perusal of the literature reveals no report on the manganese(II) ditolyldithiophosphates. In continuation of our earlier work on ditolyldithiophosphates derivatives, we report in this paper the synthesis and characterization of manganese(II) ditolyldithiophosphates and their adducts with nitrogen and phosphorus donor bases like 1,10-phenanthroline, N_2_C_12_H_8_, 2,2′-bipyridyl, N_2_C_10_H_8_, pyridine, NC_5_H_5_, and triphenylphosphine, P(C_6_H_5_)_3_. The electrochemistry of manganese complexes has also been investigated. The complexes were also screened for their antimicrobial activities against some bacteria and fungus.

## 2. Experimental

### 2.1. Materials and Methods

Stringent precautions were taken to exclude moisture. Moisture was carefully excluded throughout the experimental manipulations by using standard Schlenk techniques. *Ortho*-, *meta*-, and *para*hydroxytoluene (cresols) were distilled before use. Manganese dichloride tetrahydrate (Thomas baker) was used as received. Sodium salts of *O*,*O*′-(*o*-, *m*-, *p*- and *p*-Cl-*m*-ditolyl)dithiophosphoric acids were prepared by the literature method [29]. The solvents used, benzene, acetone, dichloromethane, hexane, and ethanol were purified and dried by standard methods before use.

### 2.2. Physical Measurements

Chlorine was estimated volumetrically by Volhard's method [30]. Manganese was estimated gravimetrically as Mn_2_P_2_O_7_ [30]. IR spectra were recorded in KBr pellets in the range of 4000–200 cm^−1^ with a Perkin Elmer-spectrum RX1 FT-IR spectrophotometer and Shimadzu FT-IR-8400S. The electronic spectra of the complexes were recorded in a range of 200–800 nm on a T90 + UV/VIS spectrophotometer using a pair of matched quartz cells of 10 mm path length at an ambient temperature. The mass spectra were recorded on VG-70-S mass spectrophotometer. The room temperature magnetic susceptibility measurements have been carried out by vibrating sample magnetometer (VSM). Molar conductance of the complexes was determined by using digital conductivity meter Century CC 601. The thermograms were analyzed by using Perkin Elmer, diamond TG/DTA instrument. Recrystallized alumina sample holder was used with the heating rate of 20°C per minute. The thermograms were recorded in the temperature range from 30°C to 1000°C. The experiment was carried out under a flow rate of 50 mL per minute of nitrogen atmosphere. The cyclic voltammograms were recorded on Autolab. The potential is applied between the reference electrode (Ag/AgCl) and the working electrode (Gold electrode), and the current is measured between the working electrode and the counter electrode (platinum wire). For all the measurements, 0.1 M phosphate buffer solution (pH = 7) was used. Elemental analyses (C, H, and N) were carried out on Vario EL III and CHNS-932 Leco Elemental analyser; their results were found to be in good agreement (±0.3%) with the calculated values.

### 2.3. Synthesis of [{(o-CH_3_C_6_H_4_O)_2_PS_2_}_2_Mn] (**1**)

An Acetone solution (30 mL) of MnCl_2_.4H_2_O (0.30 g, 1.51 mmol) was added dropwise to acetone solution of (*o*-CH_3_C_6_H_4_O)_2_PS_2_Na (1.00 g, 3.00 mmol) with constant stirring and then refluxed for 4 h. The color of contents changed to colorless with the precipitation of sodium chloride. The precipitates of sodium chloride were filtered off by using a funnel fitted with G-4 disc. Excess of solvent from the filtrate was evaporated in *vacuo* which resulted in the formation of the complex [{(*o*-CH_3_C_6_H_4_O)_2_PS_2_}_2_Mn] (**1**) as white solid in 95% yield. The complexes **2–4** were prepared by similar procedure.

### 2.4. Synthesis of [{(o-CH_3_C_6_H_4_O)_2_PS_2_}_2_Mn·N_2_C_12_H_8_] (**5**)

A mixture of MnCl_2_.4H_2_O (0.30 g, 1.51 mmol) and N_2_C_12_H_8_ (0.27 g, 1.49 mmol) in water-ethanol medium (30 mL) was added to the aqueous solution of (*o*-CH_3_C_6_H_4_O)_2_PS_2_Na (1.00 g, 3.01 mmol) in a dropwise manner through a dropping funnel. Immediately, the yellow colored precipitates were formed which were separated by filteration using funnel fitted with G-4 disc. The product [{(*o*-CH_3_C_6_H_4_O)_2_PS_2_}_2_Mn·N_2_C_12_H_8_] (**5**) was obtained as yellow solid in 94% yield. Adducts numbered **6–16** were isolated by similar route.

### 2.5. Synthesis of [{(o-CH_3_C_6_H_4_O)_2_PS_2_}_2_Mn·2P(C_6_H_5_)_3_] (**17**)

Acetone solution (30 mL) of (*o*-CH_3_C_6_H_4_O)_2_PS_2_Na (1.00 g, 3.01 mmol) was added to a acetone solution (10 mL) of MnCl_2_.4H_2_O (0.30 g, 1.50 mmol) with constant stirring at room temperature followed by the addition of the solution of P(C_6_H_5_)_3_ (0.79 g, 3.01 mmol) in acetone (10 mL). The reaction mixture was refluxed for 4 h. The precipitated sodium chloride was removed by filtration using a funnel fitted with G-4 disc. The desired product [{(*o*-CH_3_C_6_H_4_O)_2_PS_2_}_2_Mn·2P(C_6_H_5_)_3_] (**17**) was obtained in 90% yield as white solid. Complexes numbered **18–20** were prepared by this method.

### 2.6. Biological Activity

#### 2.6.1. Antibacterial

Test samples were prepared in different concentrations (100, 150, 200, and 250 ppm) in DMSO. Agar medium (20 mL) was poured into each petri plate and left to solidify. The plates were then swabbed with broth cultures of the respective microorganisms (*E. coli, K. pneumonia, *and* P. aeruginosa*) and kept for 15 min for adsorption to take place. Using a punch, *≈*6 mm diameter, wells were bored in the seeded agar plates and 100 *μ*L of the DMSO solution of each test compound was added into the wells. DMSO was used as the control for all the test compounds as it exhibited no effect on the organism tested, and Ciprofloxacin was used as the standard drug. After holding the plates at room temperature for 2 h to allow diffusion of the compounds into the agar, the plates were incubated at 37°C for 24 h. The antibacterial activity was determined by measuring the diameter of the inhibition zone. The entire tests were made in triplicates, and the mean of the diameter of zone of inhibition was calculated.

#### 2.6.2. Antifungal

Potato dextrose medium (PDA) was prepared in a flask and sterilized. 100 *μ*L of each sample was added to the PDA medium and poured into each sterilized Petri plate. Mycelial discs taken from the standard culture (*S. rolfsii*) of fungi were grown on PDA medium for 7 days. These cultures were used for aseptic inoculation in the sterilized Petri dish. Standard cultures, inoculated at 28 ± 1°C, were used as control. The efficacy of each sample was determined by measuring the radial fungal growth. The radial growth of the colony was measured in two directions at right angle to each other, and the average of two replicates was recorded in each case. Data were expressed as percent inhibition over the control from the size of the colonies. The percent inhibition was calculated using the formulae: % inhibition = ((*C* − *T*)/*C*) × 100, where *C* is the diameter of the fungus colony in the control plate after 96 h incubation and *T* is the diameter of the fungus colony in the tested plate after the same incubation period. Both antibacterial and antifungal activities were tested in the Bio-assay lab, Department of Chemistry, University of Jammu, Jammu.

## 3. Results and Discussion

**Scheme 1:**
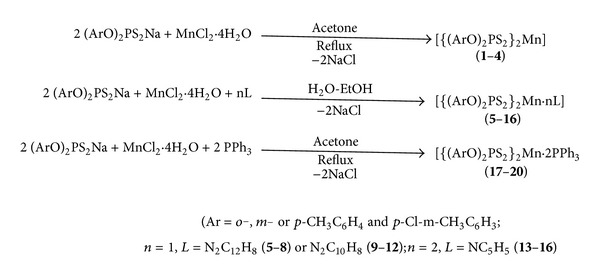
Reactions showing synthesis of *O,O*′-(*o*-, *m*-, *p*- and *p*-Cl-*m*-ditolyl) dithiophosphato complexes of Mn(II) and their adducts with nitrogen and phosphorus donor ligands.

The reactions of manganese dichloride (tetrahydrate) with sodium salt of *O,O*′-(*o*-, *m*-, *p*- and *p*-Cl-*m*-ditolyl)dithiophosphates in refluxed acetone with constant stirring in 1 : 2 molar stoichiometry yielded the complexes corresponding to [{(ArO)_2_PS_2_}_2_Mn] (**1**–**4**) (Ar = *o*-, *m*-, *p*-CH_3_C_6_H_4_ and *p*-Cl-*m*-CH_3_C_6_H_3_). The adducts were prepared by the reaction MnCl_2_·4H_2_O, [(*o*-, *m*-, *p*-CH_3_C_6_H_4_O or *p*-Cl-*m*-CH_3_C_6_H_3_O)_2_PS_2_Na] and donor base in 1 : 2 : 1 and 1 : 2 : 2 molar stoichiometry for bidentate (N_2_C_12_H_8_ (**5**–**8**) and N_2_C_10_H_8_ (**9**–**12**)) and monodentate donor ligand, (NC_5_H_5_ (**13**–**16**) and P(C_6_H_5_)_3_ (**17**–**20**)), respectively (Scheme 1). It is pertinent to mention that adducts of manganese ditolyldithiophosphates with nitrogen donors were prepared in water-ethanol medium, while adducts with triphenylphosphine were prepared in acetone. This choice of solvent medium was made on the basis of better solvation of triphenylphosphine in acetone in comparison to water-ethanol medium.

All these complexes are soluble in common organic solvents and insoluble in solvents like n-hexane and carbon tetrachloride. The complexes (**1**–**4**) obtained are white solids, whereas adducts with nitrogen donors are yellow (**5**–**16**), and that of phosphorus donor are white (**17**–**20**) solids. The synthetic and analytical data of the complexes (**1**–**20**) are summarized in Table 1.

### 3.1. IR Spectra

IR spectral assignments of these complexes (**1**–**20**) were made on the basis of relevant literature reports [26–28, 31, 32]. The comparison of IR spectra of these complexes with starting materials has also shown significant and characteristic changes and also shifting of bands. Two strong intensity bands were observed in the region 1160.7–1080.0 cm^−1^ and 960.2–896.8 cm^−1^, which may be ascribed to the **ν**(P)−O−C and **ν**P−O−(C) vibrations, respectively. The bands for **ν**P=S and **ν**P−S were observed in the regions 696.2–651.8 cm^−1^ and 640.3–541.9 cm^−1^. The bands due to **ν**P=S and **ν**P−S vibrations have depicted a shift of 10–30 cm^−1^ towards the lower frequency region in comparison to the parent dithiophosphate ligands. The observation of two closely spaced bands arising from *v*(PS_2_) vibrations is entirely typical for bidentate chelating dithiophosphates units. This shift of **ν**P−S vibrations is, perhaps, due to bidentate mode of bonding by dithiophosphates ligands. The presence of a band in the region 291.0–214.3 cm^−1^, attributed to **ν**Mn−S, is indicative of the formation of manganese-sulfur bond. This is supported by the literature survey [26]. Cavell et al. have reported similar structure IR frequency ranges for Mn-S bond which is in agreement with our observations. IR spectra have also revealed the presence of bands of **ν**Mn-N and **ν**Mn-P in the regions 486.0–422.3 cm^−1^ and 384.9–360.2 cm^−1^, respectively [31, 32]. The relevant IR spectral data of these complexes are given in Table 2.

### 3.2. Mass Spectra

The mass spectra of a few representative complexes (**1, 5**, **8, 9, 11, 14, 16, 17** and **19**) have exhibited the presence of molecular ion peak. In addition to the molecular ion peak, several other peaks were also observed, which are corresponding to the fragmented species after the consecutive removal of different groups. The occurrence of molecular ion peak in the complexes is supporting the monomeric nature of the complexes. Furthermore, the adducts (**8** and **16**) that contain multiple chlorines (i.e., two chlorine atoms) also show isotopic peaks. Based on the presence of the peaks in the mass spectra of some of the representative complexes, the various fragments have been given in Table 3.

### 3.3. Electronic Spectra

The intense electronic spectra of the complexes (**1**–**20**) show absorption in the ranges of 339–396 nm, 431–479 nm, and 510–573 nm, which may be ascribed to ^6^A_1g_ → ^4^E_g_ (^4^D), ^6^A_1g_ → ^4^E_g_ (^4^G) and ^6^A_1g_ → ^4^T_1g_ (^4^G) and are assigned to *d-d* transition [33]. The transition in these complexes indicated that the manganese is *d* ^5^ with the ground term ^6^ *S* which do not split and transformed to the ^6^A_1g_ state. The transition in these complexes is both spin forbidden and orbital forbidden, due to this, the absorption bands are extremely weak. Other weak intensity absorption bands in the region 247–296 nm are observed in adducts (**5–20**) which may be ascribed to the transition between the metal to ligand (M→L) charge transfer spectra [34]. This signifies that there is another moiety present in these adducts that is, donor atoms of 1,10-phenanthroline, 2,2′-bipyridyl, pyridine, and triphenylphosphine. The tentative assignments of the important bands for the complexes **(1**–**4)** and adducts **(5**–**20)** have been made and summarized in Table 4.

### 3.4. Magnetic Susceptibility

As a consequence of the weak field nature of the dithiophosphate ligand, effective magnetic moment values (*μ*_eff_) of all the manganese(II) complexes are high and appear in the range of 5.60–5.90 B.M. Magnetic susceptibility measurements show that these complexes are paramagnetic [26, 27]. The relevant magnetic moment values of these complexes are tabulated in Table 4.

### 3.5. Molar Conductance

The mononuclear complexes are dissolved in DMSO, and the molar conductance of 10^-3 ^M solutions at 30°C was measured. It is concluded from the results that the complexes have a molar conductance in the range of 4.36–9.81 Ω^−1^cm^2 ^mol^−1^, which indicates the nonionic nature of these complexes [6]. Hence, these complexes have nonelectrolytic properties in DMSO. The molar conductance value of the complexes is given in Table 1.

### 3.6. Thermogravimetric Analysis

**Figure 1:**
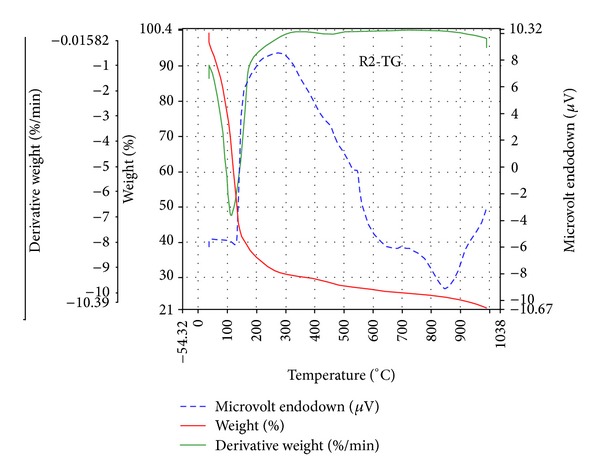
TGA curve of complex [{(*o-*CH_3_C_6_H_4_O)_2_PS_2_}_2_Mn] (**1**).

**Figure 2:**
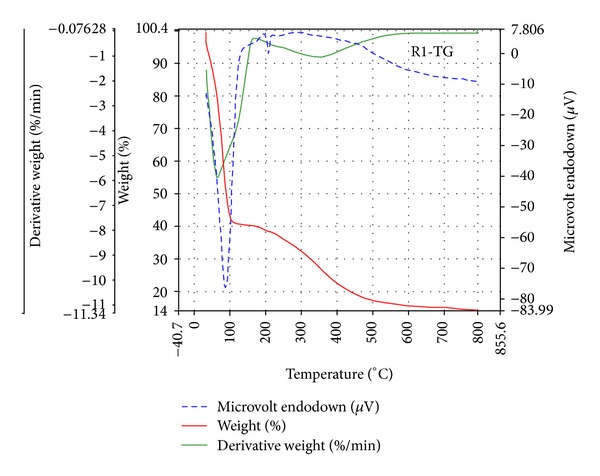
TGA curve of [{(*o-*CH_3_C_6_H_4_O)_2_PS_2_}_2_Mn·N_2_C_12_H_8_] (**5**).

The thermal behaviour of the complex [{(*o-*CH_3_C_6_H_4_O)_2_PS_2_}_2_Mn] (**1**) displayed a thermolysis step that covers a temperature range from 150 to 986°C. The main weight loss occurs in the steeply descending segment of the TG curve (Figure 1); the DTG curve shows the increasing rate of the weight loss up to 986.71°C. This weight loss of 58.58% at 151.7°C is due to the formation of bis(dithiometaphosphato) manganese(II) [Mn(S_2_PO)_2_], (the calculated weight loss is 58.86%) as an intermediate product, which agrees with thermogravimetric data for dithiophosphates. The weight of the final residue is 21.38% of the initial weight of the sample; this corresponds to the formation of MnSO_4_ contaminated with other thermolysis products (the calculated value is 22.42%) at 986.71°C. The thermogravimetric analysis of the adduct [{(*o-*CH_3_C_6_H_4_O)_2_PS_2_}_2_Mn·N_2_C_12_H_8_] (**5**) (Figure 2) exhibited loss of the donor ligand resulting in the formation of the intermediate compound [{(*o-*CH_3_C_6_H_4_O)_2_PS_2_}_2_Mn] (the calculated weight loss is 21.85%) at 68.99°C. The rate of the weight loss begins to increase steeply at 303.92°C. The weight loss at this point is due to the formation of [Mn(S_2_PO)_2_] (the calculated weight loss is 67.85%). The weight of the final residue is 14.14% of the initial weight of the sample; this corresponds to the formation of MnS contaminated with other thermolysis products (the calculated value is 10.09%) at 788.99°C.

The DSC curve for the complex [{(*o-*CH_3_C_6_H_4_O)_2_PS_2_}_2_Mn] shows an endotherm in the region of stability of [Mn(S_2_PO)_2_] near 151.7°C. Two endotherms are observed for the adduct [{(*o-*CH_3_C_6_H_4_O)_2_PS_2_}_2_Mn·N_2_C_12_H_8_], one sharp endotherm (extrapolated to 68.99°C) corresponds to the weight loss leading to the formation of [{(*o-*CH_3_C_6_H_4_O)_2_PS_2_}_2_Mn], and a less intense endotherm appears in the region of formation of [Mn(S_2_PO)_2_].

### 3.7. Cyclic Voltammetry

**Figure 3:**
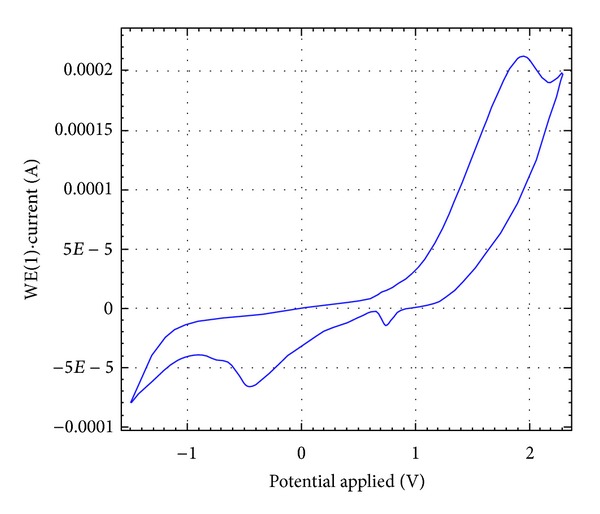
Cyclic voltammetric curve of complex [{(*o*-CH_3_C_6_H_4_O)_2_PS_2_}_2_Mn·2P(C_6_H_5_)_3_] (**17**).

The complex [{(*o*-CH_3_C_6_H_4_O)_2_PS_2_}_2_Mn·2P(C_6_H_5_)_3_] (**17**) is electroactive with respect to the metal center. The cyclic voltammogram (Figure 3) of the complex in the potential range of +2.5 to −1.5 V at a scan rate of 100 mV/s exhibited two redox processes; each reduction is associated with a single-electron transfer process. Two well-defined one-electron cyclic responses were observed as discussed in Table 5. The first reduction peak was observed at *E*_*pc*_ = 1.878 V with the cathodic peak current, *i*_*c*_ = 5.56 × 10^−5^ A, and a corresponding oxidation peak at *E*_*pa*_ = −0.465 V with the anodic peak current, *i*_*c*_ = −2.27 × 10^−5^ A. The second redox process was related with the reduction peak at *E*_*pc*_ = −1.127 V with the cathodic peak current, *i*_*c*_ = −3.19 × 10^−5^ A, with a corresponding oxidation peak at *E*_*pa*_ = 0.733 V with the anodic peak current, *i*_*c*_ = −1.2 × 10^−5^ A.

### 3.8. Antimicrobial Screening

#### 3.8.1. Antibacterial

**Figure 4:**
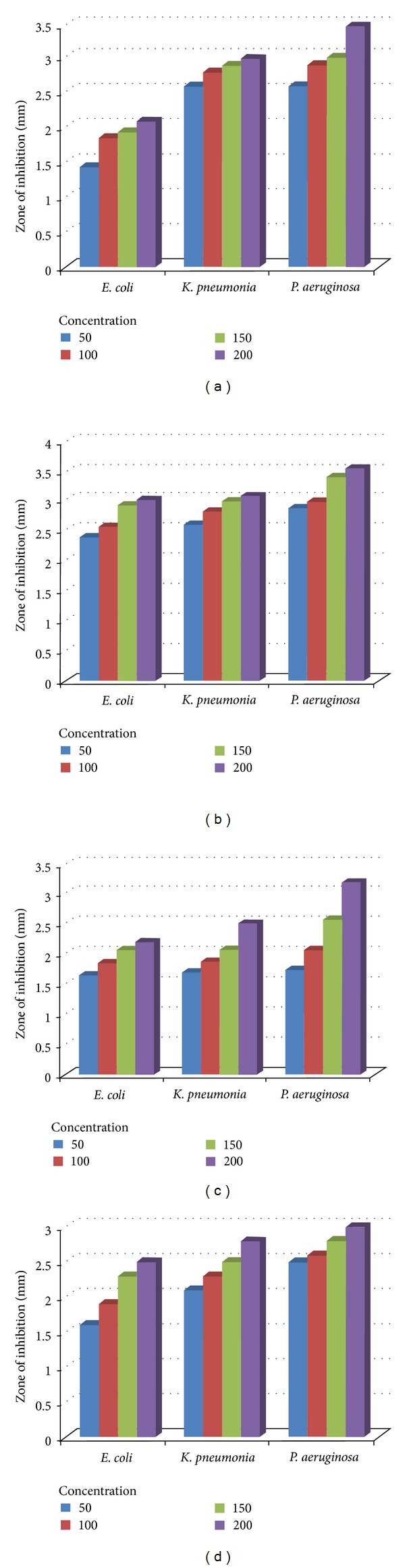
Comparative results of antibacterial screening data for: (a) Ciprofloxacin (drug), (b) MnCl_2_·4H_2_O, (c) [{(*o*-CH_3_C_6_H_4_O)_2_PS_2_}_2_Mn] (**1**), and (d) [{(*o*-CH_3_C_6_H_4_O)_2_PS_2_}_2_Mn·N_2_C_12_H_8_] (**5**).

The bacterial species under investigation were the strains of three human pathogenic gram negative bacteria: *Escherichia coli, Klebsiella pneumonia, *and* Pseudomonas aeruginosa. *The free ligands, MnCl_2_·4H_2_O, and a few complexes were screened for their *in vitro* antibacterial study by well diffusion method [35]. The results achieved by these studies have been enlisted in Table 6. The antibacterial data reveals that MnCl_2_·4H_2_O in solution shows prominent inhibition capacities while the free ligands did not possess antibacterial properties against the bacterial species under study. The results also confirmed that the antimicrobial activity of the metal chelates shows more inhibitory effects than the parent ligand. Furthermore, the [{(*o-*CH_3_C_6_H_4_O)_2_PS_2_}_2_Mn·N_2_C_12_H_8_] (**5**) exhibited enhanced activity than the [{(*o-*CH_3_C_6_H_4_O)_2_PS_2_}_2_Mn] (**1**). On chelation, the polarity of the metal ion will reduce to a greater extent due to the overlap of the ligand orbital and partial sharing of the positive charge of the metal ion with donor groups [36]. Further, it increases the delocalization of *π*-electrons around the whole chelate ring and enhances the penetration of the complexes into lipid membranes and blocking of the metal binding sites in the enzymes of microorganisms. These complexes also disturb the respiration process of the cell and thus block the synthesis of proteins, which restricts further growth of the organisms. It has also been observed that concentration plays a vital role in increasing the degree of inhibition. The inhibition is directly proportional to the concentration. The bacterial growth inhibitory capacity of ligand and its complexes with manganese(II) have also been illustrated in Figures 4(a)–4(d).

#### 3.8.2. Antifungal

**Figure 5:**
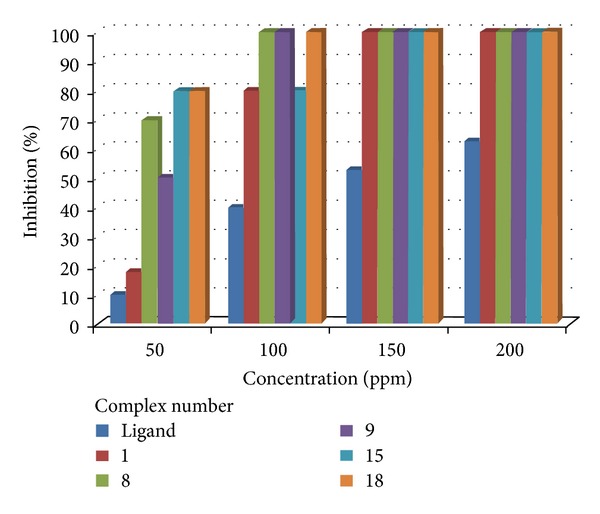
Comparative results of antifungal screening data.

The antifungal activity of ligands and a few representative metal complexes were evaluated by the poisoned food technique [35] against plant pathogenic strain, *S. rolfsii*. The antifungal screening data are given in Table 7, which shows that the colony diameter of the fungus decreases as the concentration of the complex increases; that is, all the complexes inhibited the growth of fungus significantly. This shows a linear relationship between concentration and percent inhibition. The increase in antimicrobial activity is due to faster diffusion of metal complexes as a whole through the cell membrane or due to combined activity effect of the metal and the ligand. It is evident from the antifungal screening data that adducts of nitrogen and phosphorus donor ligands (**8**, **9**, **15**, and **18**) are more potent than the parent complex **1**. The chelation theory accounts for the increased activity of the metal complexes [36]. The results of fungi-toxicity analysis have been illustrated in Figure 5. Both antibacterial and antifungal activities were tested in the Bio-assay lab, Department of Chemistry, University of Jammu, Jammu.

### 3.9. Structural Features

The outcome of the above results confirms the formation of the manganese(II) ditolyldithiophosphate complexes as indicated from elemental analyses, magnetic susceptibility, molar conductance, thermogravimetric, cyclic voltammetry, and spectral analyses including ESI mass spectrometry, IR, and UV-visible. The shiftings of **ν**P=S and **ν**P−S bands (10–30 cm^−1^) in comparison to the parent dithiophosphate ligands are quite diagnostic to propose bidentate mode of bonding of dithio moiety with manganese. Further, these bands have smaller gap between each other compared to the parent dithio ligand, which is also supporting the bidentate mode of attachment. The mass spectra support the respective molecular masses and fragmentation pattern as well as monomeric nature of these complexes. The UV-visible and magnetic susceptibility analysis confirmed the high spin nature of manganese atom in these complexes.

**Figure 6:**
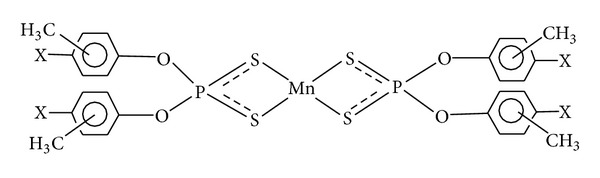
Proposed square planar structure of bis-*O,O*′-(*o-, m-, p- *and *p-*Cl*-m- *ditolyl)dithiophosphates of manganese(II) (**1**–**4**); X = H, CH_3_, or Cl.

**Figure 7:**
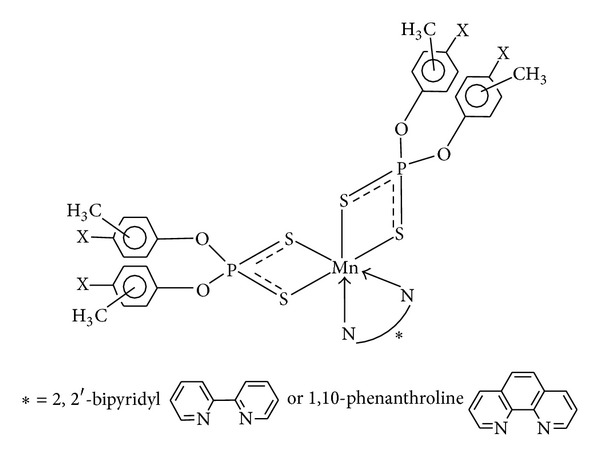
Proposed octahedral structure of manganese(II) complexes with 1,10-phenanthroline (**5**–**8**) and 2,2′-bipyridyl (**9**–**12**); X = H, CH_3_, or Cl.

**Figure 8:**
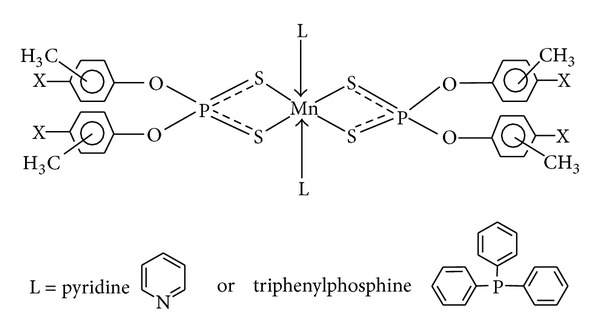
Proposed octahedral structure of complexes of manganese(II) with pyridine (**13**–**16**) and triphenylphosphine (**17**–**20**); X = H, CH_3_, or Cl.

Attempts to obtain suitable crystals for the X-ray diffraction study have so far been unsuccessful. From the above data, plausible-four and six-folded geometries may be suggested for these complexes and adducts (Figures 6, 7, and 8).

## 4. Conclusion

We have synthesized and characterized a series of twenty new *O,O*′-(*o-*, *m*-, *p-*, *p-*Cl*-m*-ditolyl) dithiophosphate derivatives of manganese. In conjunction with the literature reports and observations based on elemental analysis, IR, mass spectral studies, and thermogravimetric analysis, bidentate chelation by the ligand to the manganese(II) ion may be postulated in these complexes. The melting points were found to be consistent with the decomposition pattern in the thermogravimetric analyses. The cyclic voltammetric analysis predicted the two electron reduction of the metal. The complexes are found to have higher biological activities as compared to the respective ligand and the parent drug.

## Figures and Tables

**Table 1:** Synthetic and analytical data for the Mn(II) ditolyldithiophosphates.

S. no.	Complex	Physical state* [Yield (%)]	m.p. (°C)	Molar conductance (Ω^−1^ cm^2^ mol^−1^)	Analysis found (calcd.) (in %)					
					C	H	S	N	Cl	Mn
1.	[{(*o*-CH_3_C_6_H_4_O)_2_PS_2_}_2_Mn]	White (95)	55	4.62	49.89 (49.92)	4.17 (4.19)	19.03 (19.04)	—	—	8.13 (8.16)
2.	[{(*m*-CH_3_C_6_H_4_O)_2_PS_2_}_2_Mn]	White (94)	56	5.62	49.89 (49.92)	4.18 (4.19)	19.02 (19.04)	—	—	8.12 (8.16)
3.	[{(*p*-CH_3_C_6_H_4_O)_2_PS_2_}_2_Mn]	White (98)	55	5.30	49.90 (49.92)	4.18 (4.19)	19.02 (19.04)	—	—	8.13 (8.16)
4.	[{(*p*-Cl-*m*-CH_3_C_6_H_3_O)_2_PS_2_}_2_Mn]	White (97)	57	6.42	41.42 (41.44)	2.96 (2.98)	15.79 (15.81)	—	17.46 (17.48)	6.75 (6.77)
5.	[{(*o*-CH_3_C_6_H_4_O)_2_PS_2_}_2_Mn · N_2_C_12_H_8_]	Yellow (94)	61	7.63	56.23 (56.26)	4.24 (4.25)	15.00 (15.02)	3.26 (3.28)	—	6.40 (6.43)
6.	[{(*m*-CH_3_C_6_H_4_O)_2_PS_2_}_2_Mn · N_2_C_12_H_8_]	Yellow (92)	63	7.63	56.24 (56.26)	4.22 (4.25)	15.00 (15.02)	3.25 (3.28)	—	6.41 (6.43)
7.	[{(*p*-CH_3_C_6_H_4_O)_2_PS_2_}_2_Mn · N_2_C_12_H_8_]	Yellow (97)	62	6.54	56.24 (56.26)	4.22 (4.25)	14.99 (15.02)	3.27 (3.28)	—	6.40 (6.43)
8.	[{(*p*-Cl-*m*-CH_3_C_6_H_3_O)_2_PS_2_}_2_Mn · N_2_C_12_H_8_]	Yellow (98)	63	9.81	48.43 (48.45)	3.22 (3.25)	12.90 (12.93)	3.22 (3.25)	14.28 (14.30)	5.51 (5.54)
9.	[{(*o*-CH_3_C_6_H_4_O)_2_PS_2_}_2_Mn · N_2_C_10_H_8_]	Yellow (96)	60	4.36	54.99 (55.00)	4.35 (4.37)	15.43 (15.46)	3.37 (3.38)	—	6.60 (6.62)
10.	[{(*m*-CH_3_C_6_H_4_O)_2_PS_2_}_2_Mn · N_2_C_10_H_8_]	Yellow (91)	61	7.63	54.97 (55.00)	4.34 (4.37)	15.44 (15.46)	3.36 (3.38)	—	6.59 (6.62)
11.	[{(*p*-CH_3_C_6_H_4_O)_2_PS_2_}_2_Mn · N_2_C_10_H_8_]	Yellow (97)	60	5.45	54.97 (55.00)	4.36 (4.37)	15.43 (15.46)	3.37 (3.38)	—	6.59 (6.62)
12.	[{(*p*-Cl-*m*-CH_3_C_6_H_3_O)_2_PS_2_}_2_Mn · N_2_C_10_H_8_]	Yellow (98)	62	5.45	47.15 (47.17)	3.27 (3.30)	13.23 (13.26)	2.89 (2.90)	14.63 (14.66)	5.66 (5.68)
13.	[{(*o*-CH_3_C_6_H_4_O)_2_PS_2_}_2_Mn · 2NC_5_H_5_]	Pale yellow (92)	56	5.45	54.84 (54.87)	4.57 (4.60)	15.41 (15.42)	3.34 (3.37)	—	6.58 (6.60)
14.	[{(*m*-CH_3_C_6_H_4_O)_2_PS_2_}_2_Mn · 2NC_5_H_5_]	Pale yellow (90)	57	6.54	54.85 (54.87)	4.58 (4.60)	15.40 (15.42)	3.35 (3.37)	—	6.59 (6.60)
15.	[{(*p*-CH_3_C_6_H_4_O)_2_PS_2_}_2_Mn · 2NC_5_H_5_]	Pale yellow (95)	56	4.36	54.86 (54.87)	4.57 (4.60)	15.40 (15.42)	3.36 (3.37)	—	6.57 (6.60)
16.	[{(*p*-Cl-*m*-CH_3_C_6_H_3_O)_2_PS_2_}_2_Mn · 2NC_5_H_5_]	Pale yellow (96)	58	4.36	47.06 (47.07)	3.50 (3.53)	13.20 (13.23)	2.87 (2.89)	14.60 (14.63)	5.66 (5.67)
17.	[{(*o*-CH_3_C_6_H_4_O)_2_PS_2_}_2_Mn · 2P(C_6_H_5_)_3_]	White (90)	64	6.54	64.13 (64.15)	4.85 (4.88)	10.68 (10.70)	—	—	4.56 (4.58)
18.	[{(*m*-CH_3_C_6_H_4_O)_2_PS_2_}_2_Mn · 2P(C_6_H_5_)_3_]	White (90)	63	6.54	64.13 (64.15)	4.85 (4.88)	10.67 (10.70)	—	—	4.56 (4.58)
19.	[{(*p*-CH_3_C_6_H_4_O)_2_PS_2_}_2_Mn · 2P(C_6_H_5_)_3_]	White (94)	67	5.45	64.12 (64.15)	4.86 (4.88)	10.68 (10.70)	—	—	4.57 (4.58)
20.	[{(*p*-Cl-*m*-CH_3_C_6_H_3_O)_2_PS_2_}_2_Mn · 2P(C_6_H_5_)_3_]	White (96)	62	6.54	57.52 (57.54)	4.05 (4.07)	9.58 (9.60)	—	10.59 (10.61)	4.10 (4.11)

∗: solid.

**Table 2:** IR spectral data for Mn(II) ditolyldithiophosphates (cm^−1^).

S. no.	*v*(P)–O–C	*v*P–O–(C)	*v*P=S	*v*P–S	*v*Mn–S	*v*Mn–P	*v*Mn–N
1.	1159.2, s	923.4, s	686.3, s	546.8, m	235.6, w	—	—
2.	1109.1, s	919.2, s	686.4, s	581.4, m	249.5, w	—	—
3.	1103.2, s	925.7, s	696.0, s	561.7, m	247.3, w	—	—
4.	1140.3, s	957.0, s	684.8, s	607.6, m	246.8, w	—	—
5.	1143.7, s	900.7, s	682.7, s	551.6, m	252.4, w	—	445.5, w
6.	1121.4, s	903.6, s	675.2, s	586.4, m	273.6, w	—	453.6, w
7.	1112.7, s	898.7, s	692.4, s	565.4, m	254.9, w	—	476.3, w
8.	1149.5, s	954.7, s	688.5, s	640.3, m	263.4, w	—	445.5, w
9.	1157.1, s	906.4, s	669.2, s	552.4, m	273.4, w	—	472.6, w
10.	1110.9, s	900.3, s	671.1, s	566.4, m	261.2, w	—	428.1, w
11.	1101.2, s	896.8, s	694.3, s	560.1, m	254.9, w	—	464.8, w
12.	1151.4, s	954.7, s	651.8, s	609.4, m	255.7, w	—	484.1, w
13.	1108.9, s	910.3, s	671.1, s	559.3, m	232.7, w	—	441.6, w
14.	1080.0, s	919.9, s	696.2, s	590.1, m	245.7, w	—	486.0, w
15.	1103.2, s	910.3, s	694.3, s	592.4, m	250.0, w	—	422.3, w
16.	1159.3, s	960.2, s	683.6, s	601.3, m	246.3, w	—	442.6,w
17.	1105.1, s	908.4, s	673.7, s	541.9, m	214.3, w	384.9, w	—
18.	1103.2, s	925.4, s	692.4, s	587.6, m	242.4, w	379.4, w	—
19.	1123.2, s	902.6, s	692.4, s	557.4, m	291.0, w	361.1, w	—
20.	1160.7, s	957.4, s	680.1, s	603.5, m	282.4, w	360.2, w	—

s: strong; m: medium; w: weak.

**Table 3:** Mass spectral data of the Mn(II) ditolyldithiophosphates.

S. no.	M. Wt.	*m*/*z*, relative intensities of the ions and assignment
1.	673.6	[M^+^] 673.6 (10) [{(*o*-CH_3_C_6_H_4_O)_2_PS_2_}_2_Mn]^+^; [M^+^] 364.3 (14) [{(*o*-CH_3_C_6_H_4_O)_2_PS_2_}Mn]^+^; [M^+^] 277.1 (45) [Mn(S_2_PO)_2_]^+^; [M^+^] 107.1 (59) [*o*-CH_3_C_6_H_4_O]^−^

5.	853.8	[M^+^] 853.8 (12) [{(*p*-CH_3_C_6_H_4_O)_2_PS_2_}_2_Mn · C_12_H_8_N_2_]^+^; [M^+^] 544.5 (18) [{(*p*-CH_3_C_6_H_4_O)_2_PS_2_}Mn · C_12_H_8_N_2_]^+^; [M^+^] 309.3 (56) [(*p*-CH_3_C_6_H_4_O)_2_PS_2_]^−^; [M^+^] 218.2 (67) [(*p*-CH_3_C_6_H_4_O)P(O)S_2_]^−^; [M^+^] 107.1 (70) [*p*-CH_3_C_6_H_4_O]^−^

8.	991.6	[M^+^]^†^ 991.6 (15), 993.6 (9), 995.6 (5) [{(*p*-Cl-*m*-CH_3_C_6_H_3_O)_2_PS_2_}_2_Mn · C_12_H_8_N_2_]^+^ [M^+^]^†^ 788.4 (17), 790.4 (11), 792.4 (9) [{(*p*-Cl-*m*-CH_3_C_6_H_3_O)_2_PS_2_}Mn · C_12_H_8_N_2_]^+^; [M^+^]^†^ 433.1 (21), 433.3 (14), 433.5 (10) [{(*p*-Cl-*m*-CH_3_C_6_H_3_O)_2_PS_2_}Mn] [M^+^] 277.2 (48) [(C_6_H_3_O)_2_PS_2_]^−^; [M^+^] 201.2 (56) [*m*-CH_3_C_6_H_3_OPS_2_]^−^; [M^+^]^†^ 141.5 (60), 143.5 (32), 145.5 (17) [*p*-Cl-*m*-CH_3_C_6_H_3_O]^−^;

9.	829.8	[M^+^] 829.8 (7) [{(*o*-CH_3_C_6_H_4_O)_2_PS_2_}_2_Mn · C_10_H_8_N_2_]^+^; [M^+^] 520.4 (98) [{(*o*-CH_3_C_6_H_4_O)_2_PS_2_}Mn · C_10_H_8_N_2_]^+^; [M^+^] 364.3 (14) [{(*o*-CH_3_C_6_H_4_O)_2_PS_2_}Mn]^+^; [M^+^] 277.1 (7) [Mn(S_2_PO)_2_]^+^; [M^+^] 107.1 (20) [*o*-CH_3_C_6_H_4_O]^−^;

11.	829.8	[M^+^] 829.8 (8) [{(*p*-CH_3_C_6_H_4_O)_2_PS_2_}_2_Mn · C_10_H_8_N_2_]^+^; [M^+^] 520.4 (98) [{(*p*-CH_3_C_6_H_4_O)_2_PS_2_}Mn · C_10_H_8_N_2_]^+^; [M^+^] 364.3 (14) [{(*p*-CH_3_C_6_H_4_O)_2_PS_2_}Mn]^+^; [M^+^] 277.1 (7) [Mn(S_2_PO)_2_]^+^; [M^+^] 107.1 (20) [*p*-CH_3_C_6_H_4_O]^−^;

14.	831.8	[M^+^] 831.8 (7) [{(*m-*CH_3_C_6_H_4_O)_2_PS_2_}_2_Mn · 2C_5_H_5_N]^+^; [M^+^] 364.3 (98) [{(*m*-CH_3_C_6_H_4_O)_2_PS_2_}Mn]^+^; [M^+^] 309.3 (40) [(*m*-CH_3_C_6_H_4_O)_2_PS_2_]^−^; [M^+^] 202.5 (40) [*m*-CH_3_C_6_H_4_OPS_2_]^−^; [M^+^] 277.1 (7) [Mn(S_2_PO)_2_]^+^; [M^+^] 107.1 (20) [*m*-CH_3_C_6_H_4_O]^−^

16.	969.6	[M^+^]^†^ 969.6 (12) 971.6 (8), 973.6 (3) [{(*p*-Cl-*m*-CH_3_C_6_H_3_O)_2_PS_2_}_2_Mn · 2C_5_H_5_N]^+^; [M^+^]^†^ 512.2 (18), 514.2 (11), 516.2 (6) [(*p*-Cl-*m*-CH_3_C_6_H_3_O)_2_PS_2_Mn · C_5_H_5_N]^+^;[M^+^] 277.2 (45) [(C_6_H_3_O)_2_PS_2_]^−^; [M^+^] 201.2 (56) [*m*-CH_3_C_6_H_3_OPS_2_]^−^; [M^+^]^†^ 141.5 (65), 143.5 (34), 145.5 (19) [*p*-Cl-*m*-CH_3_C_6_H_3_O]^−^; [M^+^] 106.1 (91) [*m*-CH_3_C_6_H_3_O]^−^;

17.	1198.2	[M^+^] 1198.2 (6) [{(*o*-CH_3_C_6_H_4_O)_2_PS_2_}_2_Mn · 2P(C_6_H_5_)_3_]^+^; [M^+^] 364.3 (10) [{(*o*-CH_3_C_6_H_4_O)_2_PS_2_}Mn]^+^; [M^+^] 202.2 (18) [*o*-CH_3_C_6_H_4_OPS_2_]^−^; [M^+^] 277.1 (7) [Mn(S_2_PO)_2_]^+^; [M^+^] 107.1 (20) [*o*-CH_3_C_6_H_4_O]^−^;

19.	1198.2	[M^+^] 1198.2 (8) [{(*p*-CH_3_C_6_H_4_O)_2_PS_2_}_2_Mn · 2P(C_6_H_5_)_3_]^+^; [M^+^] 364.3 (10) [{(*p*-CH_3_C_6_H_4_O)_2_PS_2_}Mn]^+^; [M^+^] 278.7 (98) [(*p*-C_6_H_4_O)_2_PS_2_]^−^; [M^+^] 202.2 (18) [*p*-CH_3_C_6_H_4_OPS_2_]^−^; [M^+^] 107.1 (20) [*p*-CH_3_C_6_H_4_O]^−^;

^†^isotopic peaks.

**Table 4:** Magnetic moment and electronic spectral data for the Mn(II) ditolyldithiophosphates.

S. no.	*μ*_eff_	*λ*_max⁡_ (nm)	Assignments
1.	—	347	^ 6^A_1g_ → ^4^E_g_ (^4^D)
		431	^ 6^A_1g_ → ^4^E_g_ (^4^G)
		510	^ 6^A_1g_ → ^4^T_1g_ (^4^G)

2.	—	339	^ 6^A_1g_ → ^4^E_g_ (^4^D)
		435	^ 6^A_1g_ → ^4^E_g_ (^4^G)
		512	^ 6^A_1g_ → ^4^T_1g_ (^4^G)

3.	—	344	^ 6^A_1g_ → ^4^E_g_ (^4^D)
		433	^ 6^A_1g_ → ^4^E_g_ (^4^G)
		517	^ 6^A_1g_ → ^4^T_1g_ (^4^G)

4.	5.90	341	^ 6^A_1g_ → ^4^E_g_ (^4^D)
		432	^ 6^A_1g_ → ^4^E_g_ (^4^G)
		514	^ 6^A_1g_ → ^4^T_1g_ (^4^G)

5.	—	370	^ 6^A_1g_ → ^4^E_g_ (^4^D)
		449	^ 6^A_1g_ → ^4^E_g_ (^4^G)
		536	^ 6^A_1g_ → ^4^T_1g_ (^4^G)
		269, 286	Charge transfer

6.	5.60	365	^ 6^A_1g_ → ^4^E_g_ (^4^D)
		452	^ 6^A_1g_ → ^4^E_g_ (^4^G)
		542	^ 6^A_1g_ → ^4^T_1g_ (^4^G)
		271, 285	Charge transfer

7.	—	373	^ 6^A_1g_ → ^4^E_g_ (^4^D)
		456	^ 6^A_1g_ → ^4^E_g_ (^4^G)
		538	^ 6^A_1g_ → ^4^T_1g_ (^4^G)
		264, 286	Charge transfer

8.	—	364	^ 6^A_1g_ → ^4^E_g_ (^4^D)
		465	^ 6^A_1g_ → ^4^E_g_ (^4^G)
		532	^ 6^A_1g_ → ^4^T_1g_ (^4^G)
		264, 284	Charge transfer

9.	5.68	363	^ 6^A_1g_ → ^4^E_g_ (^4^D)
		450	^ 6^A_1g_ → ^4^E_g_ (^4^G)
		540	^ 6^A_1g_ → ^4^T_1g_ (^4^G)
		271, 283	Charge transfer

10.	—	361	^ 6^A_1g_ → ^4^E_g_ (^4^D)
		446	^ 6^A_1g_ → ^4^E_g_ (^4^G)
		536	^ 6^A_1g_ → ^4^T_1g_ (^4^G)
		266, 285	Charge transfer

11.	—	359	^ 6^A_1g_ → ^4^E_g_ (^4^D)
		445	^ 6^A_1g_ → ^4^E_g_ (^4^G)
		533	^ 6^A_1g_ → ^4^T_1g_ (^4^G)
		270, 282	Charge transfer

12.	—	370	^ 6^A_1g_ → ^4^E_g_ (^4^D)
		453	^ 6^A_1g_ → ^4^E_g_ (^4^G)
		539	^ 6^A_1g_ → ^4^T_1g_ (^4^G)
		269, 280	Charge transfer

13.	—	396	^ 6^A_1g_ → ^4^E_g_ (^4^D)
		479	^ 6^A_1g_ → ^4^E_g_ (^4^G)
		542	^ 6^A_1g_ → ^4^T_1g_ (^4^G)
		273, 294	Charge transfer

14.	—	390	^ 6^A_1g_ → ^4^E_g_ (^4^D)
		472	^ 6^A_1g_ → ^4^E_g_ (^4^G)
		573	^ 6^A_1g_ → ^4^T_1g_ (^4^G)
		270, 296	Charge transfer

15.	—	393	^ 6^A_1g_ → ^4^E_g_ (^4^D)
		478	^ 6^A_1g_ → ^4^E_g_ (^4^G)
		549	^ 6^A_1g_ → ^4^T_1g_ (^4^G)
		273, 294	Charge transfer

16.	5.65	390	^ 6^A_1g_ → ^4^E_g_ (^4^D)
		470	^ 6^A_1g_ → ^4^E_g_ (^4^G)
		565	^ 6^A_1g_ → ^4^T_1g_ (^4^G)
		277, 295	Charge transfer

17.	—	352	^ 6^A_1g_ → ^4^E_g_ (^4^D)
		435	^ 6^A_1g_ → ^4^E_g_ (^4^G)
		524	^ 6^A_1g_ → ^4^T_1g_ (^4^G)
		253, 276	Charge transfer

18.	—	356	^ 6^A_1g_ → ^4^E_g_ (^4^D)
		447	^ 6^A_1g_ → ^4^E_g_ (^4^G)
		521	^ 6^A_1g_ → ^4^T_1g_ (^4^G)
		247, 274	Charge transfer

19.	5.76	353	^ 6^A_1g_ → ^4^E_g_ (^4^D)
		432	^ 6^A_1g_ → ^4^E_g_ (^4^G)
		525	^ 6^A_1g_ → ^4^T_1g_ (^4^G)
		251, 273	Charge transfer

20.	—	356	^ 6^A_1g_ → ^4^E_g_ (^4^D)
		442	^ 6^A_1g_ → ^4^E_g_ (^4^G)
		528	^ 6^A_1g_ → ^4^T_1g_ (^4^G)
		249, 275	Charge transfer

**Table 5:** Cyclic voltammetric analysis data of complex [{(*o*-CH_3_C_6_H_4_O)_2_PS_2_}_2_Mn · 2P(C_6_H_5_)_3_] (**17**).

Index	Peak position	Peak height	Base start	Base end	Peak (1/2)
1	1.8784	5.56 × 10^−5^	1.3754	2.2739	0.29762
2	0.73334	−1.22 × 10^−5^	0.64056	0.91644	−0.07277
3	−1.127	3.19 × 10^−5^	−1.4835	−0.46295	0.25486
4	−0.46539	−2.27 × 10^−5^	−0.7193	−0.16754	−0.17766

**Table 6:** Antibacterial activities of the Mn(II) ditolyldithiophosphates (zone of inhibition, in mm).

Compound	Concentration (ppm)	Bacterial species		
		*Escherichia coli *	*Klebsiella pneumonia *	*Pseudomonas aeruginosa *
Ciprofloxacin (standard drug)	50	1.4	2.5	2.6
	100	1.8	2.7	2.9
	150	1.9	2.8	3.0
	200	2.1	2.9	3.5

MnCl_2_ · 4H_2_O (metal salt)	50	2.4	2.6	2.9
	100	2.6	2.8	3.0
	150	2.9	3.0	3.4
	200	3.0	3.1	3.6

Free ligand (*o*-CH_3_C_6_H_4_O)_2_PS_2_Na	50	0.0	0.0	0.0
	100	0.0	0.0	0.0
	150	0.0	0.0	0.0
	200	0.0	0.0	0.0

(**1**)	50	1.7	1.7	1.8
	100	1.9	1.9	2.1
	150	2.1	2.1	2.6
	200	2.3	2.5	3.2

(**5**)	50	1.6	2.1	2.5
	100	1.9	2.3	2.6
	150	2.3	2.5	2.8
	200	2.5	2.8	3.0

**Table 7:** *In vitro* evaluation of Mn(II) ditolyldithiophosphates against *Sclerotium rolfsii*.

S. no.	Concentration (ppm)	Colony diameter (*T*) (in mm)	% inhibition *I* = [(*C* − *T*)/*C*] × 100
Free ligand (*o*-CH_3_C_6_H_4_O)_2_PS_2_Na	50	36	10
	100	24	40
	150	19	52.5
	200	15	62.5

**1**	50	41	18
	100	10	80
	150	0	100
	200	0	100

**8**	50	15	70
	100	0	100
	150	0	100
	200	0	100

**9**	50	25	50
	100	0	100
	150	0	100
	200	0	100

**15**	50	10	80
	100	10	80
	150	0	100
	200	0	100

**18**	50	10	80
	100	0	100
	150	0	100
	200	0	100

Mean colony diameter of control (C) = 50 mm.
